# Fungi–Nematode Interactions: Diversity, Ecology, and Biocontrol Prospects in Agriculture

**DOI:** 10.3390/jof6040206

**Published:** 2020-10-04

**Authors:** Ying Zhang, Shuoshuo Li, Haixia Li, Ruirui Wang, Ke-Qin Zhang, Jianping Xu

**Affiliations:** 1 State Key Laboratory for Conservation and Utilization of Bio-Resources in Yunnan, and Key Laboratory for Southwest Microbial Diversity of the Ministry of Education, Yunnan University, Kunming 650032, China; yzh_1210@hotmail.com (Y.Z.); Li50522@163.com (S.L.); 17806253435@163.com (H.L.); Wrui931017@163.com (R.W.); 2 School of Life Science, Yunnan University, Kunming 650032, China; 3 Department of Biology, McMaster University, Hamilton, ON L8S 4K1, Canada

**Keywords:** nematophagous fungi, cross-kingdom interactions, food-web cycling, phytophagous nematodes, soilborne fungal pathogens

## Abstract

Fungi and nematodes are among the most abundant organisms in soil habitats. They provide essential ecosystem services and play crucial roles for maintaining the stability of food-webs and for facilitating nutrient cycling. As two of the very abundant groups of organisms, fungi and nematodes interact with each other in multiple ways. Here in this review, we provide a broad framework of interactions between fungi and nematodes with an emphasis on those that impact crops and agriculture ecosystems. We describe the diversity and evolution of fungi that closely interact with nematodes, including food fungi for nematodes as well as fungi that feed on nematodes. Among the nematophagous fungi, those that produce specialized nematode-trapping devices are especially interesting, and a great deal is known about their diversity, evolution, and molecular mechanisms of interactions with nematodes. Some of the fungi and nematodes are significant pathogens and pests to crops. We summarize the ecological and molecular mechanisms identified so far that impact, either directly or indirectly, the interactions among phytopathogenic fungi, phytopathogenic nematodes, and crop plants. The potential applications of our understanding to controlling phytophagous nematodes and soilborne fungal pathogens in agricultural fields are discussed.

## 1. Introduction

Ecosystems consist of many types of organisms, including different types of microscopic organisms such as bacteria, archaea, protozoa, fungi, and small animals such as nematodes. Together, these organisms interact with each other and with macroscopic organisms such as plants and large animals to perform ecosystem functions. Their interactions happen in multiple ways, can be direct or indirect, involving two or more partners, and occur through different mechanisms such as predation, parasitism, mutualism, or competition. These interactions are critical for maintaining ecosystem balance [1]. 

Fungi and nematodes are among the most abundant organisms in the terrestrial ecosystem. The phylum Nematoda, also known as the roundworms, is the second largest phylum in the animal kingdom, encompassing an estimated 500,000 species [2]. Ninety percent of terrestrial nematodes reside in the top 15 cm of soil, and they play an important role in the nitrogen cycle by way of nitrogen mineralization. Nematodes do not decompose organic matter but instead are parasitic or free-living organisms that feed on living materials [3]. On the other hand, fungi are the principal decomposers of dead organic matter; they perform fundamental roles in nutrient cycling in the ecosystem. Although fungi may look like plants, they are in fact evolutionarily more closely related to animals than to plants. Both fungi and nematodes (as well as all animals) are heterotrophs. They are commonly found co-existing in a diversity of natural and man-made ecosystems, especially in the rhizosphere of plants, including crops, with significant impacts on agriculture and forestry. Consequently, interactions among fungi and nematodes have attracted significant attention.

**Figure 1:**
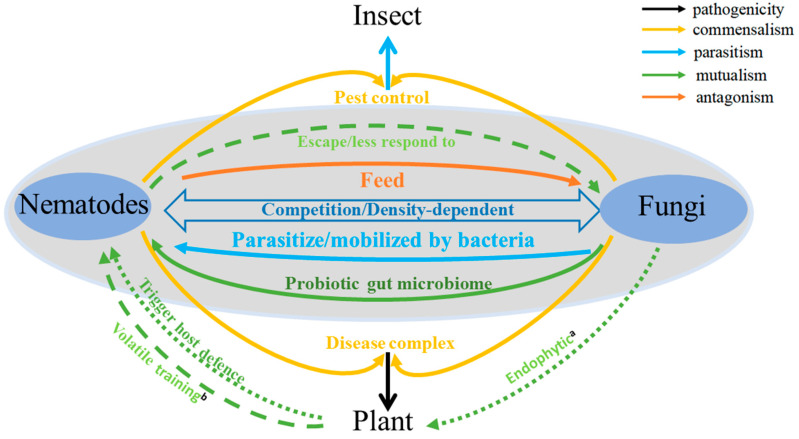
Fungi-nematode interactions in soil. a: Endophytic fungi trigger host plant defense against plant pathogenic nematodes (PPNs). b: Plants help nematodes escape fungal attacks through volatile training.

Nematode and fungi arose about 550–600 mya and 1050 mya, respectively. They likely co-existed and interacted with each other in soils before plants colonized terrestrial habitats about 450 mya [4,5]. The co-existence and interactions between nematodes and fungi, whether antagonistic or mutualistic, direct or indirect, are fundamental for understanding their ecosystem effects and their potential manipulations in agriculture. An important long-term goal in agriculture pest and pathogen management is to identify novel control strategies against phytophagous nematodes and soilborne fungal pathogens, to help increase both the quality and quantity of agricultural products. In this review, we summarize our current knowledge of the interactions between nematodes and fungi. Specifically, we focus on the interactions between these two groups of organisms that have shown both antagonistic and mutualistic interactions to each other, either directly or indirectly (Figure 1). We note that the nature of their interactions can vary greatly among the different fungal and nematode species. Furthermore, the interactions between two organisms are not static but can be impacted by environmental factors to influence both the type and magnitude of their interactions [6].

## 2. Antagonistic Interactions

Antagonistic interactions between fungi and nematodes are as numerous as they are varied. For example, many nematodes, such as *Aphelenchus avenae*, *Aphelenchoides* spp., and *Paraphelenchus acontioides*, can feed on a diversity of fungi. These are commonly referred to as fungivorous nematodes [7]. In contrast, a number of fungal species such as *Arthrobotrys oligospora* can prey on nematodes and their eggs, consuming them as food. Such fungi are known as nematophagous fungi [8].

### 2.1. Nematodes Feeding on and Antagonizing Fungi

Many of the nematodes have fungi in their diets or feed exclusively on fungi [9]. Thus, as a major component of the soil food web, nematodes can influence both the fungal diversity and abundance and community structure, including crop growth and tolerance to soil pollution. Fungivorous nematodes commonly exist in soil containing many different fungal species. Nematodes in the genera *Aphelenchus*, *Aphelenchoides*, *Ditylenchus*, and *Tylenchus* are among the most common fungivorous nematodes [10]. Generally, fungivorous nematodes feed on a diversity of soil fungi, including saprophytic, plant-pathogenic, and plant-beneficial (such as mycorrhizal) fungi and are known as polyphagous nematodes [11]. While the population densities of fungivorous nematodes in soil may be lower than those of phytoparasitic nematodes and bacterivorous nematodes, the population densities of fungivorous nematodes can increase rapidly in the presence of suitable fungal food [10]. Depending on the soil microbiome, nematode feeding on soil fungi could have significant impacts on soil ecology and crop productivity. For example, if fungivorous nematodes were to feed on plant-pathogenic fungi, the phytopathogen population in the soil could be suppressed. However, if mycopathogenic fungi (e.g., species in the genera *Gliocladium* and *Trichoderma*) antagonistic to plant-pathogenic fungi are found to be the food of nematodes, then the beneficial effects of these antagonist fungi to plants would be reduced due to the actions of these nematodes. All these fungi with different relationships to nematodes and to each other can be present in the same ecological niches. In addition, the food fungi for nematodes are not all identical. Different food fungi may present different attractiveness to the fungivorous nematodes and that attractiveness may vary depending on the environmental conditions. Furthermore, both fungi and nematodes are mobile, in different ways, to allow them to disperse across ecological niches [2,9].

One group of fungi that nematodes like to feed on is the mycorrhizal fungi. Indeed, the interactions between mycorrhizal fungi and fungivorous nematodes have been the subject of intensive investigations because of the potential effects of grazing on the function of the mycorrhiza in nutrient uptake and growth of the host plants. Indeed, surveys have found that fungal fruiting bodies (mushrooms) produced by ectomycorrhizal fungi often contain nematodes. Such fungal grazing by nematodes can have other effects, such as the release of nutrients immobilized in fungal biomass as resources for bacteria [12]. Aside from ectomycorrhizal fungi, endomycorrhizal fungi also interact with nematodes. For example, the reproduction of *Aphelenchoides* sp. nematodes can be triggered by the co-inoculation of arbuscular mycorrhizal (AM) fungi, which lead plants to achieve further growth and greater arsenic (As) tolerance at low As-polluted soil. [13]. This could have significant implications for changing the composition and infectivity of field assemblages of AM fungi. On the other hand, nematodes of the genus *Aphelenchus* can prevent the symbiosis between endomycorrhizal fungi of the genus *Glomus* with pine roots. In such instances, fumigant nematicides need to be used to disinfest the soil before pine seedling planting can be successful. Indeed, fumigation not only increases the endomycorrhizal infection of pine roots but also enables the pine trees to utilize the dead nematodes as an excellent pabulum [6].

Fungivorous nematodes could be multifunctional. *A. avenae* is a non-parasitic fungivorous nematode that can control plant-pathogenic fungi [7]. For example, both *A. avenae* and *Aphelenchoides* spp. suppressed *Rhizoctonia solani* and reduced the damping of disease in cauliflower seedlings [14]. In addition, *A. avenae* can suppress the propagation of the plant parasitic nematode *Ditylenchus destructor,* suggesting that it is a potential biocontrol agent against both certain plant-pathogenic fungi and plant parasitic nematodes [15]. Genetic analyses of cell wall-degrading enzymes from *A. avenae* support the roles of these enzymes in feeding on both plant pathogenic fungi and a plant parasitic nematode [16]. Interestingly, in the pinewood nematode *Bursaphelenchus xylophilus*, a cellulase similar to those in fungi and associated with the ability to parasitize living plants was identified as most likely the result of horizontal gene transfer, acquired during its evolution of plant parasitism [17].

In the fungal prey–nematode predator relationship, just like other types of prey–predator relationships, the fungal prey can develop resistance mechanisms against nematode predation. One type of fungal prey defense is the production and secretion of toxic secondary metabolites and toxic proteins [18]. For example, the model mushroom *Coprinopsis cinerea* produces a toxic substance on its mycelial surface that can kill nematodes upon contact [19]. Indeed, upon predation by nematodes, *C. cinerea* exhibited a comprehensive set of differentially expressed genes (DEGs) and the production of a bacterial cytolysin-like toxin. Some of these DEGs in *C. cinerea* represent a novel type of fungal effector protein against nematodes [20].

### 2.2. Fungi Antagonizing Nematodes

The above examples show nematodes antagonizing and feeding on fungi; the opposite can also happen and is known to be quite common in nature. The interaction between nematophagous fungi and nematodes has played a crucial role in understanding broad fungi–nematode interactions. Nematophagous fungi, including those that are variously called predaceous, nematode-trapping, and nematode-destroying fungi, possess amazing abilities to capture nematodes and reduce the population size of plant-parasitic nematodes. Such abilities have significant applied interests in agriculture [21,22]. Studies of nematophagous fungi and their interactions with nematodes have revealed several mechanisms of their interactions at the molecular, cellular, organismal, and ecological levels. Indeed, such studies have propelled their interactions as models for studying inter-kingdom interactions in predator–prey coevolution, and for biocontrol applications [23,24,25,26,27,28,29].

#### 2.2.1. Diversity and Evolution of Fungal Predation Structures

Nematophagous fungi have been traditionally divided into four main groups based on the mechanisms that they use to attack nematodes: (i) nematode-trapping fungi, producing extensive hyphal networks, knobs, and constricting rings as trapping devices to catch and hold live nematodes; (ii) endoparasitic fungi, as obligate parasites that exist as conidia in the environment and infect nematodes by either adhering to the surface of the prey or by directly being ingested by the nematodes followed by germination, growth, and nematode killing; (iii) egg- and cyst-parasitic fungi, as facultative parasites that grow on and parasitize the sedentary stages of nematodes such as eggs; and (iv) toxin-producing fungi, producing toxic compounds that are active against nematodes [30,31]. Except for the egg stage, most nematodes at other life stages are capable of moving through their environments, which presents a challenge for relatively slow-growing and immobile fungal parasites. However, many fungi have evolved to parasitize mobile stages of nematodes by employing complex and sophisticated predation structures, including (1) trapping structures to immobilize nematodes; (2) adhesive conidia to attach and colonize the nematodes’ pseudocoeloms; (3) acanthocytes, spiny balls, and stephanocysts to damage the cuticle of nematodes and then consume them; and (4) gun cells to launch finger-like tubes directly at the target nematodes [8,21,32].

The interaction between nematophagous fungi and nematodes induces morphogenesis and virulence gene expression in these fungi, signaling a transition from their saprobic stage to phagocytic stage. Evolutionarily, nematophagous fungi are widely distributed across many phylogenetically-independent taxonomic groups, indicating that the ability to phagocytize nematodes has evolved multiple times [23,33]. Among the nematode-trapping fungi, there are also multiple types of trapping structures, including constricting rings and five types of adhesive traps (sessile adhesive knobs, stalked adhesive knobs, adhesive nets, adhesive columns, and non-constricting rings), all of which were originated from the vegetative hyphae [34]. Consistent with frequent and independent origins of nematode-trapping devices, members of the Orbiliaceae produce five types of traps, among them *Arthrobotrys dactyloides, Arthrobotrys superba, Arthrobotrys oligospora*, and *Monacrosporium gephyropagum* are capable of forming conidial traps—traps formed directly from the asexual spore, the conidia. At a low nutrient level, competition for nutrients among microorganisms can be intense; thus, the ability of fungal spores to directly germinate into the traps could be highly advantageous [35,36]. Consistent with convergent evolution in some trapping structures, two groups of fungi from two different phyla, namely *Zoophagus* species of Zygomycota and *Nematoctonus* species of Basidiomycota, can both produce adhesive knobs [37]. However, traps based on adhesive hyphae are restricted to the fungal genera *Stylopage* and *Cystopage* of Zygomycetes [38], while the prominent fungi parasitizing cyst nematode juveniles, *Hirsutella rhossiliensis* and *Hirsutella minnesotensis*, are representatives of adhesive spores [30]. Some species of endoparasites have developed morphologically-adapted conidia that, when eaten by the nematodes, become lodged in either its buccal cavity or esophagus. These species belong almost exclusively to the genus *Harposporium* [39]. Among other nematode-trapping fungal structures, stephanocysts are restricted to the genus *Hyphoderma* of Basidiomycota [40]. Spiny balls and acanthocytes are known only by *Coprinus comatus* and *Stropharia rugosoannulata*, respectively, in Agaricales of Basidiomycota [41,42]. Finally, a very peculiar attack device called the “gun cell” is produced by endoparasitic fungi in the genus *Haptoglossa* (Oomycete fungi) [43].

Among the broad groups of nematophagous fungi, those that form specialized morphological adaptations to capture nematodes are especially interesting. These nematode-trapping fungi (NTF) can switch their lifestyle from saprophytes to predators under certain cues. Such transitions have made them good models for studying inter-kingdom communication with regard to the mechanisms of fungal pathogenesis and adaptation [23,27,44]. In recent years, -omics studies have significantly improved our understanding of host–microbe interactions, especially in those cases where the microorganisms are difficult to grow under laboratory conditions [45]. In the case of fungi–nematode interactions, sequencing of the genomes of the nematode female and egg parasite *Pochonia chlamydosporia* [46]; the nematode-trapping fungi *Arthrobotrys oligospora* [44], *Monacrosporium haptotylum* [25], and *Drechslerella stenobrocha* [47]; and the facultative nematode endoparasite *H. minnesotensis* have greatly contributed to our understanding of the evolutionarily distinct strategies of fungal pathogenesis against nematodes [48].

Using NTF in the phylum Ascomycota as models, phylogenies based on genes and genomes from both the nuclei and the mitochondria support that, within the Orbiliales, the nematode-trapping mechanisms have evolved along two major lineages. In one lineage, the species form constricting rings. In the second, the species form adhesive traps, including three-dimensional hyphal networks, adhesive hyphal branches, and adhesive knobs [23,33,49]. Furthermore, a combined five-gene phylogeny and molecular clock calibration based on two fossil records revealed that the organismic interactions between NTF and nematodes likely dates back to more than 419 million years of co-evolution, with the active carnivores (fungi with constricting rings) and passive carnivores (fungi with adhesive traps) diverged from each other around 246 Mya, shortly after the occurrence of the Permian–Triassic extinction event about 251.4 Mya [23]. However, no major carnivorous ascomycete divergence has been correlated to the Cretaceous–Tertiary extinction event. More research is needed to identify if the evolution of fungal carnivorism was a response to mass extinction events.

Despite the diverse morphogenesis, different kinds of nematode traps share two structural features that are different from vegetative hyphae. The first one is the presence of numerous cytosolic organelles, commonly known as dense bodies [50]. These dense bodies are peroxisomal in nature and only detected in nematode-trapping fungi, but not in endoparasitic nematophagous fungi that infected their host with adhesive or non-adhesive spores [51]. Their functions seemed to be involved in adhering to nematodes and supplying energy and/or structural components to the invading hyphae [51]. A recent study showed that disruption of the gene *Aoime2* caused reductions in both trap formation and electron-dense bodies in trap cells [52], with substantially fewer nematodes captured by the mutants. The second feature common to the adhesive traps (columns, networks, and knobs) is the presence of extensive layers of extracellular polymers, which are thought to be important for adhesion of the traps to the surface of nematodes [53]. Recent genome comparisons and surface structural analyses revealed evidence for expansion of adhesion genes in NTF genomes and with associated increase in trap surface adhesiveness. Both of these can enhance the ability of the fungi to penetrate and digest the nematodes and likely represent the key drivers of fungal adaptation in trapping nematodes [27].

#### 2.2.2. Host Recognition, Adhesion, Host Specificity, and Infection Process

As a shared characteristic among all types of nematophagous fungi, recognition of the hosts and adhesion to the cuticle of the nematodes or eggshells by the fungi are the first steps in infection. The nematode cuticle is a solid exoskeleton that mainly consists of proteins. The exoskeleton acts as a barrier against environment stresses and potential pathogen attacks [54]. At present, how NTF penetrates the nematode exoskeleton has not been fully elucidated. Current research results suggest that secreted enzymes from NTF play a major role during invasion of the nematodes by the fungi. Specifically, genetic, ultrastructural, and histochemical studies showed that the presence of extracellular hydrolytic enzymes such as chitinases, collagenases, and proteases are essential for nematode cuticle penetration [55]. Indeed, phylogenetic analysis of the pathogenicity-related serine proteases from nematophagous and entomopathogenic fungi showed that they evolved from a common ancestor [56]. Penetration is typically followed by content digestion, resulting in the formation of a new fungal biomass inside and later outside the nematodes. Table 1 shows the four main steps of infection from the four main groups of nematophagous fungi, plus the producers of special attack devices (structures which mechanically damage the cuticle of nematodes, as the fifth group) [21,30].

Nematode-trapping fungi (NTF) are usually not host specific and can trap many types of soil-dwelling nematodes [80]. In contrast, there is some host specificity among endoparasitic fungi. The endoparasitic fungi are obligate parasites and mostly exist as conidia in the environment. The conidial attachment to a particular nematode species does not always lead to infection, but specific recognition signals for adhesion are required, as shown by the endoparasitic fungus *Drechmeria coniospora* [71,81]. Fungi that parasitize nematodes are common soil saprophytes, attacking primarily the sedentary stages (female and egg stages) of nematodes or sedentary nematodes, such as *Heterodera*, *Globodera*, and *Meloidogyne* [30]. Nematode-toxic fungi have nematode-immobilizing activity and can kill their nematode hosts by producing toxins. The success and efficiency of nematode attacks by producers of special attacking devices are also sometimes linked to the toxins produced [41]. Special attacking devices are similar to a sharp sword or acanthocytes, spiny balls, and stephanocysts, like real medieval weapons, causing damage to the nematode cuticle, resulting in extravasation of the inner contents of the nematodes and allowing complete colonization of the nematode body by fungal hyphae.

#### 2.2.3. Competition between Nematode-Trapping Fungi and Nematodes

Evolutionary arms races are common between pathogens and hosts. Evidence for such arms races has been found between nematodes and NTF. In these arms races, fungal predators continuously evolve predatory strategies to secure food from nematodes. In turn, the prey nematodes evolve counter measures, such as enhanced innate immunity and sophisticated nervous systems to sense and avoid their predator fungi. Many factors can influence such arms races. For example, in soil environments, the populations of NTF and their target nematodes not only interact with each other as predators and prey but also with other fungi and nematodes nearby, respectively. In addition, biotic factors such as other microbes and plants as well as abiotic factors such as nutrient levels can also influence NTF–nematode interactions. Systematic studies on the bitrophic (NTF and nematode) or multitrophic (plant, soil microorganisms, nematode, and NTF) interactions under natural conditions are required to obtain a broad understanding of the factors influencing the ecology and evolution of such interactions. Below we summarize our current understanding of the potential mechanisms involved in the arms race between NTFs and nematodes.

##### Innate Immune Defense Responses in Nematodes

The epidermis and the collagen-rich cuticle that surrounds the nematode provide a physical barrier to fungal pathogens. Nematodes can also sense and defend against fungal pathogens using strategies such as producing antimicrobial peptides regulated by the innate immunity system. To cope with bacterial and fungal pathogen attacks from the intestine or the cuticle, the innate immune response of *Caenorhabditis elegans* is accompanied by an increase of reactive oxygen species (ROS) [82]. The nematode genomes also contain many antimicrobial peptide (AMP)-coding genes that play important roles in their innate immunity. In one study, when *C. elegans* was infected, one of the AMPs, NLP-31, showed strong activities against several fungi, including *Drechmeria coniospora*, *Neurospora crassa*, and *Aspergillus fumigatus* [83]. The recent expansion of the AMP-encoding *nlp* genes as revealed by genome sequencing, together with the evidence for their in vivo role and the signatures of positive selection of the *nlp29* gene cluster, suggest that these genes are important for the survival of *C. elegans* when they interact with *D. coniospora* spores [84]. The FOXO transcription factor DAF-16, which lies downstream of the conserved insulin/IGF-1 signaling (IIS) pathway, is required for survival after fungal infection and wounding [85]. RNA-seq analysis further identified shared and unique signaling pathways regulated by DAF-16/FOXO and highlighted the intestinal DAF-16 regulatory components and roles of the innate immune system countering fungal pathogenesis [86].

##### Competition between Different Fungal Species and Nematodes

To survive and reproduce, nematodes and NTF need to successfully cope with many stressors and competing demands in soil. Competition can be among different fungal predators, among different nematodes, and between NTF and nematodes [87]. Surveys have found that multiple NTF species often coexist in the same niche, suggesting that they likely compete for the same prey in their natural environments. For example, *Arthrobotrys* species are sympatrically distributed and are generalist predators of nematodes. Two species in *Arthrobotrys*, namely *A. thaumasia* and *A. musiformis*, are sympatric with nematodes in more than 63% surveyed natural sites. In addition, the ability to sense prey among wild isolates of *Arthrobotrys oligospora* varied greatly [28]. Some nematodes are trapped/colonized by more than one NTF at the same time (e.g., colonized from opposite ends of the nematodes). In some of those cases, evidence for competition between NTFs has been found. For example, the hyphae of *A. oligospora* were often observed to be dead or degenerated when placed in close proximity to live mycelia of the endoparasitic fungus *D. coniospora*, consistent with the latter being an antagonist against *A. oligospora* under the specific conditions [88]. El-Borai et al. [89] indicated that the tested nematodes were repelled by activated *Arthrobotrys* species but were attracted to activated endoparasitic fungi from the genera *Myzocytium* and *Catenaria*.

Antagonistic interactions between NTF and nematodes have been detected in the soil environments. As expected, density-dependent parasitism has been reported, demonstrating that an increase in NTF density would lead to a decrease in nematode prey density, which subsequently would lead to a decrease in NTF density and an increase in nematode density. This negative frequency-dependent selection between NTF and nematodes regulates the densities of both groups of organisms [90]. This model successfully described changes in parasitism of the nematode *Heterodera schachtii* by the nematophagous fungus *Hirsutella rhossiliensis* as a function of host density, with the disease dynamics in soil microcosms exhibiting both a temporal density-dependent parasitism and a host threshold density [91]. Suppression of the root-knot nematode *Meloidogyne javanica* by NTFs *Monacrosporium cionopagum* and *H. rhossiliensis* was positively related to the nematode host *Steinenema glaseri* density, and the dynamics of suppression varied among different species [92]. In addition, spatial sampling of the nematophagous fungus *H. rhossiliensis* revealed a relationship between numbers of hosts (*Criconentella xenoplax*) and the degree of parasitism [93], with evidence of the two interacting partners possessing similar density-dependent dynamics among tested patches of agricultural fields [94]. Recent greenhouse trials also showed that parasitism of *H. rhossiliensis* was strongly correlated with the density of the soybean cyst nematode [30]. At broader geographic scales, in a survey of 53 citrus orchards in central ridge and flatwood ecoregions of Florida, the spatial patterns of entomopathogenic nematode species were found to be modulated by variations in their susceptibilities to nematophagous fungal species (*Catenaria* sp., *A. musiformis, Arthrobotrys dactyloides, Paecilomyces lilacinus, A. oligospora*, and *Gamsylella gephyropaga*) across habitats [95]. However, strong and diverse top-down control effects on the nematode community in coastal sand dunes were found in a recent study, where three microbial enemies of nematodes (*Catenaria* spp., *H. rhossiliensis*, and *Pasteuria penetrans*) were correlated, either positively or negatively, with plant parasitic nematode population size [96]. Together, these results are consistent with some species-specific effects for both the fungal and the nematode partners in natural and agricultural ecosystems. Aside from these individualized surveys, metagenomic methods have also been used to analyze the relationships between fungi and nematodes (as well as bacteria) in field settings [97] and revealed a diversity of spatial associations similar to those described above between plant parasitic nematodes and NTFs [98,99].

## 3. Synergistic Interactions between Phytophagous Nematodes and Phytopathogenic Fungi against Host Plants

### 3.1. Interactions between Phytophagous Nematodes and Soil-borne Fungal Pathogens

In the soil environment, opportunities exist for interactions between soil-borne pathogens and pests of plants when they occupy the same ecological niche. While antagonism can occur between them in their competitions for space and resources, synergistic interactions between them are also possible to cause greater damage to plants, including crops. For example, in the rhizosphere, nematode attacks can lower the resistance of plants to pathogens and increase their susceptibility to infection by soil-borne fungal pathogens. In these situations, the physiological states of all three interacting partners play a very important role in the outcome of such tripartite interactions.

The first example of a nematode–fungi disease complex in plants was described by Atkinson, in 1892, where he reported that the fusarium wilt of cotton (caused by *Fusarium oxysporum* f. sp. *vasinfectum*) was more severe in the presence of root-knot nematodes (*Meloidogyne* spp.) [100]. Subsequently, many other cases of synergistic interactions between nematodes and fungi have been reported, involving sedentary endoparasitic root-knot and cyst nematodes, and increasing disease severity caused by *Fusarium* or *Verticillium* wilt fungi. *Meloidogyne* spp. has been shown to interact with *Fusarium* wilt to negatively impact a number of crops, with cyst nematodes acting in a similar manner to increase wilt diseases. Meanwhile, entomopathogenic nematodes and pathogenic fungi have been shown capable of generating additive interactions to increase insect pest mortality [101]. In these cases, an initial fungal infection plays a key role in weakening the larvae and increasing the pest insects’ susceptibility to nematodes by generating a stressful condition and altering the insects’ behavior [102]. Table 2 summarizes recent examples of nematode–fungi pathogen disease complexes reported in crops and insects.

### 3.2. Factors Influencing Interactions between Phytophagous Nematodes and Phytopathogenic Fungi

As shown above, the nature of interactions between phytophagous nematodes and phytopathogenic fungi varies among the different fungal and nematode species. For example, some plant pathogenic nematodes can induce physical damage, such as small wounds, to their host plants. Such wounds may allow fungal pathogens easy access to plant tissues to cause infections. Alternatively, some nematodes may induce physiological changes in their host plants, triggering changes in fungal pathogen populations around the host plants and making them more likely to increase their population size and/or pathogenicity [121]. In addition, other biotic and abiotic factors such as host plant genotype, organic matter and nutrient availability, and other microbes could all affect the outcome of infections by nematode pests and plant fungal pathogens [117].

In agriculture fields, the fungal species composition can vary, depending on whether the fields are infested by root-knot nematodes. *Fusarium oxysporum* (11%) followed by *Fusarium solani* (6%) were found to be the most frequent fungal species associated with the presence of *Meloidogyne* spp., and fungal diversity plays an important role in the interactions between host plants and soil microorganisms [111]. For example, inoculation of certain bacterial and fungal combinations together had an inhibitory effect on each other and reduced crop disease severity [112]. On the other hand, abiotic factors such as soil moisture and soil physicochemical properties also play important roles for infection by plant fungal pathogens and nematode pests [112].

Another interesting interaction between nematodes and fungi that could have important effects on agriculture is that between entomopathogenic nematodes and entomopathogenic fungi. Together, these entomopathogenic pests and pathogens could help control pest insect populations in agricultural fields. However, to realize their potential, it is important to understand the life cycles of both the entomopathogenic nematodes and entomopathogenic fungi, and to develop strategies to allow them to grow in the same ecological niches with minimal negative impacts on each other [102]. Indeed, a previous study has shown that the virulence of both the entomopathogenic nematode *Steinernema riobravae* and entomopathogenic fungus *Beauveria bassiana* against last larval instars of *Galleria mellonella* could be synergistic or additive, depending on environmental conditions and application strategies [122].

## 4. Fungi and Nematodes Interact through a Third Party

In most natural soil ecosystems, fungal species co-occur with nematodes, and both often actively interact with plants. This cross-kingdom interaction between fungi and nematodes in the plant rhizosphere is often called a tripartite interaction. Other organisms, such as bacteria, may also be involved in this network of interactions. These interactions may involve direct cell–cell contacts. Alternatively, they may interact indirectly, using chemical signals. Indeed, chemical signals such as volatile organic compounds released by organisms such as bacteria, nematodes, fungi, or plants have been detected to initiate interactions between fungi and nematodes. Due to the ubiquitous distributions of these organisms in natural environments and agricultural fields, their interactions have significant ecological and economic impacts. Thus, it is important to develop a comprehensive understanding of such interactions involving all partners in terrestrial and agricultural ecosystems.

### 4.1. Induced Resistance

There is a broad range of detrimental microbes and nematodes that can challenge the plant’s capability for growth and survival. However, their effects on plants vary depending on other microbes in the same ecological niches. For example, colonization of plant roots by beneficial endophytic and mycorrhizal fungi can protect plants against a wide range of plant-parasitic nematodes through plant mediated mechanisms [123,124]. One example of a beneficial endophyte is the fungus *Trichoderma harzianum*. This fungus can induce jasmonic acid (JA)- and salicylic acid (SA)-regulated defense pathways in tomato (*Solanum lycopersicum*), causing resistance to the root-knot nematode *Meloidogyne incognita*. Similarly, mycorrhizal fungi represent an inextricable part of almost every plant system. Their role in suppression of plant pathogenic nematodes (PPNS) has been extensively studied and reviewed [125]. For example, plants associated with arbuscular mycorrhizal fungi (AMF) showed decreased damages caused by sedentary endoparasites than those without AMF colonization. The antagonistic action of mycorrhizal fungi against PPNS may be achieved directly, e.g., by competition for nutrients and space, or indirectly, by increasing plant tolerance, mediating induced systemic resistance (ISR) in plants, changing rhizosphere interactions by altering root exudations, or all of the above [1].

Interestingly, some nematophagous fungi can colonize plants as endophytes. Thus, they are also capable of mediating ISR against nematodes in situ. Most studies investigating fungi–nematode interactions are conducted outside of the plant hosts; few have considered the effects of these fungi in the context of plant endophytes. The soil borne *Phialemonium inflatum* is a known nematode egg-parasite fungus, which can secrete extracellular proteases and chitinases and significantly reduce hatching of *M. javanica* juveniles [126]. A recent study revealed a novel role for this fungus. Specifically, a foliar-isolated *P. inflatum* strain was shown to be endophytic in cotton, and part of a plant-fungal defensive symbiosis in cotton. Using a seed treatment inoculation, this isolate showed significant inhibitory effects against the root-knot nematode *M. javanica* as an endophyte in cotton. This was the first study demonstrating antagonistic effects of endophytic *P. inflatum* against root-knot nematodes [127]. Similarly, compared to treatments with only the nematode *M. incognita* or with neither the nematode nor the *A. oligospora,* treatment containing endophytic and rhizospheric populations of *A. oligospora* showed reduced nematode population size and increased defense-related enzymes in tomatoes [87]. The inoculation of *Drechslerella dactyloides* and *D. brochopaga* also significantly increased plants’ resistance against *M. incognita* [128].

Another example is an endophytic strain of *Pochonia chlamydosporia* that caused a moderately induced expression of genes involved in ISR in barley (*Hordeum vulgare*) [129]. However, in this study, the plants were not challenged with plant-parasitic nematodes to conclusively demonstrate priming for resistance to nematodes [129]. *Arabidopsis thaliana* root colonization by *P. chlamydosporia* showed modulated jasmonate signaling that resulted in accelerated flowering and improved yield [130]. Further studies showed that the effects were due to chitosan-mediated increases in root colonization by *P. chlamydosporia* [131,132]. Overall, these studies contribute to potential future applications of endophytes to increase plant tolerance/resistance to RKN.

### 4.2. Alteration of Root Exudates

Plant roots typically have a close association with mutualistic rhizosphere microorganisms. Together, they exude a wide range of both primary metabolites and secondary metabolites. Such metabolites can modify the surface properties of nematodes and affect microbial attachment to nematode surfaces [133]. In parallel, the success of the nematode infection and inhibition by the nematophagous microbes depends on how the plant roots and their associated microbes perceive the signaling molecules on the nematode surfaces. Indeed, metabolites exuded from plant roots affect not only the communication between plants and nematodes, but also the nematode–fungi interactions by modulating the surface properties of nematodes. One of the mechanisms of nematode suppression by *Fusarium* endophytes appears to be through altering root exudates [134]. A similar mechanism was proposed for AMF-mediated nematode suppression. In both tomatoes and bananas, AMF colonization of roots altered root exudates, leading to fewer nematodes penetrating AMF compared to roots with non-AMF colonization [135]. Specifically, root exudates altered by the nematophagous fungus *Pochonia indica* stimulated the hatching of mobile second-stage juveniles (J2s) that were dormant in nematode cysts [136], which subsequently caused a major inhibitory effect on the development of *Heterodera schachtii* in *Arabidopsis* roots [137]. *H. schachtii* is a plant pathogenic nematode capable of infecting more than 200 different plants including economically important crops such as sugar beets, tomatoes, bananas, cabbage, broccoli, and radish.

The modes of action of mycorrhizal fungi against PPN may be exhibited through a direct effect, by competition for nutrients and space, or indirect effect, by increasing plant tolerance, mediating ISR in plants, altering rhizosphere interactions due to changed root exudations, or all of these combined, depending on the species of both AMF and nematodes [125]. Further research on the nematode/microbial selectivity in the attachment and the influence of plants on these interactions could open up possibilities for manipulating these interactions to improve plant health.

### 4.3. Chemical Signals

In their natural habitats, nematodes and fungi exist as parts of complex multitrophic communities that depend on and communicate through elaborate networks of chemical signaling. A key feature of microbe–microbe interaction is the secretion of chemical mediators that can influence interactions involving both microbial partners and the co-occurring multicellular organisms. Both nematodes and fungi have developed elaborate communication systems that are based on secretion of chemicals, allowing intra- and inter-kingdom interactions.

The most common chemical signals in the evolution of predator–prey relationships are those related to recognitions of specific pathogens or food sources [138]. Recent studies based on the model nematode *Caenorhabditis elegans* identified ascarosides, a group of small molecules, as involved in inter-organismal communications. These chemicals play a central role in regulating nematode development and behavior [139,140]. Under nitrogen starvation, ascaroside-induced morphogens are required for *A. oligospora* to sense and initiate trap formation in response to the availability of nematode prey [24,141]. Recent studies have also characterized several other morphology-regulating arthrosporol metabolites from *A. oligospora* and identified them as important signaling cues for hyphal development, nematode attraction, and trap morphogenesis [29,62,142].

Ammonia is another molecule identified to influence interactions among multiple interacting partners. Specifically, it has been reported as an intracellular signal for altering fungal morphological switch and mediating interspecific interactions among bacteria, fungi, and nematodes [143]. Wang et al. [26] demonstrated that when bacteria were consumed by bacterivorous nematodes, urea production and release were enhanced by upregulating the arginase’s expression. The urease within the fungi eventually catabolized urea to ammonia, which initiates formation of predatory structures. Similarly, diketopiperazines (DKPs) were shown to facilitate chemotaxis of *Stenotrophomonas* bacteria towards fungal extracts, leading to bacterial biofilm formation on fungal nematode traps and enhancing fungal trapping activity against nematodes [36].

In the relationship among entomopathogenic nematodes, plant, insects, and nematophagous fungi, behavioral plasticity of entomopathogenic nematodes in response to a plant volatile organic compound (d-limonene) affected nematode–fungi interactions. Two mechanisms were suggested for their interactions. In the first, nematodes’ response to d-limonene may make them less likely to respond to other environmental stimuli, such as to attractants released by nematophagous fungi. In the second, the learned response by nematodes to plant volatiles may motivate entomopathogenic nematodes to move faster in the event of being exposed to such volatiles, potentially making it harder to catch them by fungal traps [144,145].

### 4.4. Microbiome

Due to technological advances in high-throughput DNA sequencing, more and more studies have examined structures of microbial communities (microbiomes), both in soil ecosystems and in nematodes’ guts in order to evaluate other biotic factors potentially involved in mediating interactions between nematodes and fungi [146]. These studies have revealed that while some microbes can exert antagonistic effects on both nematode pests and plant fungal pathogens, others can form mutualistic interactions with plant disease-causing agents. For example, in the root microbiomes of many plants, nematophagous fungi in genera *Clonostachys*, *Dactylellina, Purpureocillium*, *Pochonia*, and *Rhizophydium* often co-occur. However, the nematode-suppressing efficiency in such soils is often limited. Understanding the relationships among members of native anti-nematode microbiome are required for building effective approaches to develop nematode pest-suppressing soil [147]. For example, successive monoculture of soybeans clearly affected the assembly of both bacterial and fungal communities (i.e., the genera *Pseudomonas, Purpureocillium*, and *Pochonia*,) in the rhizosphere and negatively impacted the rhizosphere microbiome in its ability to suppress the soybean cyst nematodes [148]. Crop rotation may reverse such negative effects.

On the other hand, studies have revealed that the native microbiomes of nematodes carry a species-rich bacterial community dominated by Proteobacteria such as Enterobacteriaceae and members of the genera *Pseudomonas, Stenotrophomonas, Ochrobactrum,* and *Sphingomonas*. Several studies highlighted the influence of microbiota on *C. elegans* fitness, stress resistance, and resistance to pathogen infection [149]. For example, three *Pseudomonas* isolates were identified to be able to produce an anti-fungal effect in vitro and contribute to the worm’s defense against fungal pathogens in vivo [146,150]. Another study using *C. elegans* suggested that probiotic yeasts colonizing the nematode gut protected nematodes from infection with non-albicans *Candida* strains and alleviated pathogenic effects [151]. Unexpectedly, among the >5000 culturable fungal isolates obtained from the mycobiome of soybean cyst nematodes, using in vitro high-throughput screening, a large proportion of these cultured fungi showed bioactivity against nematode egg hatching or showed toxicity toward J2 stage nematodes [152]. Together, these results suggest that supplying one or a few fungi with anti-nematode activities to the soil environments are not sufficient to suppress the nematode populations.

## 5. Applications of Our Understanding in Fungi–Nematode Interactions in Agriculture: The Control of Phytophagous Nematodes and Soilborne Fungal Pathogens

The rhizosphere contains a complex of biological and ecological processes. A better understanding of fungi–nematode complexes could benefit the development of ecologically based management tools to control important plant pathogen and crop pests. Discoveries of antagonistic interactions between nematodes and some rhizospheric microorganisms can provide the basis for developing control strategies to enhance plant defense against soil-borne plant pathogens and root-knot nematode parasites, including *Meloidogyne* spp., etc. Below we discuss a few potential approaches.

### 5.1. Nematodes as Biocontrol Agents against Plant Pathogenic Fungi

Because fungal-feeding nematodes can be attracted to and actively feed on plant pathogenic fungi, these nematodes can potentially be used to reduce the load of fungal plant pathogens and minimize the effects of these fungal pathogens on crops.

Some species of the nematode genus *Aphelenchoides* feed on the cytoplasm of fungal hyphae by piercing and sucking using a strong stylet [37]. When mixed with the mycopathogenetic fungus *Trichoderma* spp., these nematodes are able to feed on two plant fungal pathogens, namely *Botrytis cinerea* and *Sclerotinia sclerotiorum* [6], and the combination of *T. harzianum* and *Aphelenchoides* nematode treatment resulted in the best disease control efficiency against fungal diseases [153]. One species in this nematode genus, *Aphelenchoides hylurgi* is able to parasitize both virulent and hypovirulent strains of the fungus *Cryphonectria parasitica*, the causal agent of chestnut blight [154]. Furthermore, the nematodes were also able to spread propagules of the hypovirulent strain, thus increasing the efficacy of biological control under field conditions. Another fungal-feeding nematode, *Aphelenchus avenae*, also showed strong abilities to reduce pathogen loads of two root-rot fungi in corn [155], *Rhizoctonia solani* and *Fusarium solani* [156,157], as well as one root-rot fungus, *Fusarium oxysporum*, in beans and peas [158]. However, fungivorous nematodes are often not discriminatory in their food fungal choice. They can also feed on fungi with potential beneficial effects to plants. For example, *Trichoderma harzianum*, an extensively studied biocontrol agent against the sclerotium-forming fungus, *Sclerotinia sclerotiorum*, is also a favorite food for the fungivorous nematode *Aphelenchoides saprophilus.* Consequently, *A. saprophilus* can reduce the biocontrol efficiency of *T. harzianum* against *S. sclerotiorum* [159].

At present, most fungivorous nematodes reported in the literature are those that are easy to propagate in large numbers and can be stored in a dormant stage (anhydrobiosis) for a relatively long time. They have so far not been extensively applied to agriculture or horticulture fields in the form of nematode applications. This is mainly due to the high costs associated with the production, storage, and distribution of fungivorous nematodes for commercial applications. One way to realize such commercial potential is to combine fungivorous nematodes with other agricultural practices such as crop rotation and the application of other biocontrol agents to reduce the costs and maximize the benefits.

### 5.2. Biocontrol of Nematodes with Nematophagous Fungi

It is estimated that, worldwide, plant parasitic nematodes (PPNs) cause a combined >$150 billion worth of damages to agriculture each year [31]. From an ecological perspective, this group of nematodes is one of many components in the ecosystem that interact with other organisms, contributing to the maintenance and stability of the soil food-web. Over the last 30 years, our understanding of microbial diversity and the multitrophic interactions that are manifested in the rhizosphere, as well as biological control systems as they apply to nematodes, has improved tremendously. Indeed, several environmentally benign strategies have been developed for PPN management. Among PPNs, the root-knot nematodes (RKNs; *Meloidogyne* spp.) represent the most severe challenges to crop production. In a summary of the biocontrol methods evaluated between 2015 and April 2020, 10 microfungi and 3 mushroom species were tested for their effectiveness in controlling RKNs [160]. However, most studies were conducted in laboratories and greenhouse settings and their efficacies in the field are not known. Converting the laboratory successes into equally effective field applications represents the next step of the challenge.

#### 5.2.1. Potential for the Discovery of Novel Candidates

It has been estimated that the number of culturable fungal species is between 2.2 and 3.8 million. On the basis of a 1:8.8 ratio between the numbers of cultured fungal species and the number of fungal operational taxonomic units estimated based on metagenome sequencing, there would be approximately 12 million fungal species on earth [161,162]. At present, only ~1.2% (140,000) of these have been described [163]. Thus, many new fungi with potential nematophagous activities await discovery. Even among the known culturable fungi, new compounds with novel mechanisms of nematode-parasite action have been continuously found. For example, in the fungus *Pleurotus ostreatus,* anthelmintic compounds were recently isolated and showed potent activity against a wide range of nematode species. It is possible that there are many novel fungi and novel fungal compounds effective at controlling parasitic nematodes of plants, animals, and humans [79].

Over the last few years, high-throughput sequencing of the universal barcode locus for fungi (18S, ITS rDNA) has revealed great potential for identifying fungi in many ecological niches. However, the fungal community comparisons between niches with different nematodes, especially those with different phytopathogenic nematodes, have been very limited. Microbiome studies of such ecological niches could help reveal the microbial diversities responsible for the differential distributions of phytopathogenic nematodes and assist in developing holistic management strategies with multi-target modes of action to control these pests. Integration of microfluidics, robotics, and machine learning technologies in interaction studies in microcosms between the microbiome and nematodes could provide novel ways to capitalize on our knowledge about the core microbiomes of pest nematodes. Such knowledge could help increase control efficiency and stress-resistance of biocontrol applications [164]. On the other hand, novel molecular markers could be developed to analyze the parasitic activities and population dynamics of nematophagous fungi. Such tools could allow us monitoring these fungi and their activities in agricultural fields.

#### 5.2.2. Development and Integration of New Methods

To achieve successful and reproducible biological control, we must understand the ecological interactions affecting the control agent and the target. Modern technologies can help us achieve such goals. For example, real-time quantitative PCR provides an effective way to quantify and track biocontrol agents after they are applied to soil [165]. Similarly, genetic modifications of the biocontrol agents could be used to help the organisms overexpress traits involved in pathogenicity or nematocidal activity [8,166].

Several studies have demonstrated the effectiveness of using combinations of treatments, including various cultural practices (like soil solarization and soil amendment), chemical nematicides, and biological agents in controlling PPN populations under various conditions [80]. These studies have revealed that soil physical chemical properties can have a significant influence on their control efficacies. Thus, attention should be paid to develop biocontrol protocols that are specific for targeting ecological niches.

The use of nematophagous fungi as endophytes, i.e., rhizosphere colonization by biocontrol agents, is a promising strategy for implementing biocontrol of plant-parasitic nematodes. Endophytes should be relatively easy to apply as inoculants to seeds or seedlings and could therefore be established in the root system before nematodes are attracted to roots [167].

Finally, the unpredictability and relatively low efficacy of nematode antagonists against PPN in field conditions are major obstacles for the application of biocontrol agents for managing plant-parasitic nematodes. Part of the reasons for the differences between laboratory-based and field-based trial results may be related to the intrinsic mechanisms regulating ecosystem stability in field conditions. Application of a large number of a specific organism would disturb the balance of interactions among organisms in native niches, with the target interaction between the applied biocontrol agent and PPN in the field not realized. Thus, understanding how organismal interactions in native niches are regulated could help us develop better applications that take into account native agricultural ecosystems to ultimately produce sustainable methods of crop protection while maintaining biodiversity. Studies that evaluate the effects of coadministration of multiple partners such as nematophagous fungi, mycoparasites of plant pathogens, and plant growth promotors could help generate significant data to allow a systems approach in developing biocontrol measures to minimize the effects of nematode pests and fungal pathogens on agricultural crops [168,169].

## Figures and Tables

**Table 1:** Infection process of nematophagous fungi.

Group	Representative Species	Host Recognition	Adhesion	Penetration	Digestion
Nematode-trapping fungi	*A. oligospora*	Mediated by lectin, proteins on the fungal surface interacting with sugar molecules on the nematode cuticle (GalNAc [57], AOL [58], AofleA [59], AoMad1 [60]), a nematode specific pheromone ascaroside [24], or olfactory mimicry that attracts nematode prey [29].	A physical contact between the trap cells and the nematodes, cell-to-cell communication [61], a group of volatile organic compounds furanone, pyrone, and maltol [62], nitrate [63] and autophagy [64] are required for switching from the saprophytic to the pathogenic stage during trap formation and adhesion.	Fungi pierce the cuticle by forming a penetration tube, with a combination of mechanical pressure and extracellular hydrolytic enzymes, such as serine proteases (PII [65], Aoz1 [55], Ac1 [66], Ds1 [67] Dv1 [68], Mlx [69], Mc1 [70]), collagenase, and chitinase [55].	Nematode content is converted to lipid droplets, these fungi obtain nutrients from the nematodes for their growth and reproduction.
Endoparasitic fungi	*Drechmeria coniospora*	Obligate parasites, using conidia that are ingested by their host, or by spores that adhere to the cuticle of the host [71].	Adhesive conidia that adhere to the nematode cuticle will form an appressorium that presses firmly against the nematode cuticle. Motile zoospores encyst on the nematode’s surface and germinate to produce the injection tube, to infect nematodes by injecting a sporidium [37].	A combination of enzymatic action and mechanical force, followed by vigorous growth of the trophic hyphae, to invade nematodes [72,73,74].	New conidiophores develop from bulbs at the tips of trophic hyphae inside the cadaver, tightly pressed to the internal surface of the cuticle, preventing leakage of host nutrients, perturbing nematode metabolism, and causing nematode death [75].
Egg- and cyst-parasitic fungi	*Pochonia chlamydosporia*	Aurovertin D showed strong toxicity and recognition of host [76].	Glycoproteins and appressoria responsible for the adhesion of conidia and hyphae to the eggshell [77].	Proteases and chitinases, e.g., PrC from *Clonostachys rosea* and Ver112 from *Lecanicillium psalliotae* [78].	Colonizes the host tissues to obtain nutrients and uses available sugars in the egg as a carbon source [46].
Toxin-producing fungi	*Pleurotus ostreatus*	Induces paralysis via the cilia of nematode sensory neurons [79].	All developmental stages of *C. elegans* are sensitive to *P. ostreatus*. Nematodes become paralyzed upon contacting the *P. ostreatus* hyphae.	Excess calcium influx and hypercontraction of the head and pharyngeal muscle cells in nematodes.	Toxins cause rapid and systemic necrosis in multiple tissues throughout the organism.
Fungi producers of special nematode-attacking devices	*Coprinus comatus;* *Stropharia rugosoannulata*	Sharp projections of the special attack devices, mechanically damage the cuticle of the nematode [41,42].	A penetration peg is formed and penetrates the nematode cuticle via mechanical forces and enzymatic activities.	Hyphae colonize the interior of the nematode and project themselves from the infected nematode.	Need toxin assistance to be successful in their nematicidal role (spiny balls).

**Table 2:** Examples of nematode–pathogen disease complexes reported in crops and insects.

Nematode	Pathogen	Crop/Insect	Reference
*Steinernema diaprepesi*	*Fusarium solani*	Wax moth, Weevil	[103]
*Heterorhabditis bacteriophora, Steinernema feltiae, S. kraussei*,	*Metarhizium anisopliae*	Black vine weevil	[104]
*H. sonorensis*	*F. oxysporum*	Corn earworm	[105]
*Meloidogyne incognita*	*F. oxysporium f.* sp. *phaseoli*	Bean	[106]
*M. incognita*	*F. oxysporium f.* sp.	Potato	[107]
*M. incognita*	*Rhizoctonia solani*	Green bean	[108]
*M. incognita*	*Phytophthora capsici*	Pepper	[109]
*M.* spp.	*F. oxysporum f.* sp. *lycopersici*	Tomato	[110]
*M.* spp.	*F. oxysporum, F. solani*	Tomato	[111]
*M. javanica*	*F. oxysporum f.* sp. *lycopersici*	Tomato	[112]
*M. incognita*	*F. solani*	Fig	[113]
*M. incognita*	*F. oxysporum f.* sp. *niveum*	Watermelon	[114,115]
*M. incognita*	*Ralstonia solanacearum, Phomopsis vexans*	Eggplant	[116]
*M. incognita*	*Alternaria dauci, Rhizoctonia solani*	Carrot	[117]
*Pratylenchus* spp., Trichodoridae, Heteroderidae	*Rhizoctonia solani*	Potato	[118]
*S. feltiae, S. carpocapsae, H. bacteriophora*,	*Aspergillus* spp., *Penicillium* spp.	Carob moth	[119]
*S. diaprepesi*	*F. solani*	Weevil	[120]
